# Surgical removal of ingested magnets in children: A retrospective clinical analysis of 16 patients and review of the literature

**DOI:** 10.1097/MD.0000000000042903

**Published:** 2025-06-13

**Authors:** Hyung Jun Kwon, Jinyoung Park

**Affiliations:** a Department of Surgery, School of Medicine, Kyungpook National University, Kyungpook National University Hospital, Daegu, South Korea.

**Keywords:** children, magnet ingestion, surgery

## Abstract

Foreign body ingestion is a common pediatric emergency. Notably, the inadvertent ingestion of magnets is frequently initially asymptomatic, only to be detected days later when gastrointestinal damage has already manifested. We conducted a retrospective review of the medical records of 16 pediatric patients who underwent surgical removal of ingested magnets at our hospital between January 2018 and December 2023. This review encompassed the patients’ demographic characteristics, the interval from ingestion to admission, clinical presentation, diagnostic techniques, the type and number of ingested magnets, location of injury, surgical interventions, and postoperative outcomes. The study sample comprised 11 boys and 5 girls, with a median age of 3.6 years (range, 1–14 years). The predominant presenting symptoms were abdominal pain (n = 6), abdominal pain accompanied by vomiting (n = 3), and a combination of fever, diarrhea, and seizure (n = 1); 6 patients were asymptomatic. Neodymium bead magnets were the most commonly ingested type, noted in 13 patients. All 16 patients underwent either laparoscopic exploration or laparotomy due to clinical deterioration or confirmed complications. For 13 patients, magnet removal with primary bowel repair was performed, whereas in 3 patients, magnet removal combined with segmental resection and primary bowel repair was necessary. Fifteen of the 16 patients recovered without complications; however, wound infections occurred in 2 patients, and 1 patient required adhesiolysis and segmental resection of the jejunum due to intestinal obstruction. One patient died of septic shock. The 15 surviving patients are currently in excellent condition. Magnet ingestion by children, particularly when multiple magnets are involved, constitutes a surgical emergency. Timely identification and prompt surgical intervention are critical to preventing severe complications. Improved educational and preventive strategies (including public awareness initiatives regarding the hazards of small magnets) are essential to reducing the frequency of these incidents.

## 1. Introduction

The ingestion of foreign bodies (FBs) is a common pediatric emergency, with magnets posing a particularly significant hazard due to their distinctive characteristics.^[1–4]^ The increasing availability of small, strong magnets in toys and household products, for example, to affix papers or pictures to metal surfaces such as whiteboards and refrigerators, has contributed to a surge in magnet ingestions, especially among children.^[5–10]^ Although a single ingested magnet typically traverses the gastrointestinal tract without complications, the ingestion of multiple magnets, or a single magnet ingested together with another metallic FB, may result in considerable morbidity.^[11]^ Furthermore, ingestion of magnets frequently goes unnoticed, as most children remain asymptomatic; in many cases, the presence of a magnet is only detected days later when gastrointestinal injury is already present.^[12]^ Consequently, prompt diagnosis and intervention are critical to preventing severe and life-threatening complications. This study examined the demographic characteristics, clinical presentations, surgical interventions, and postoperative outcomes of 16 children who underwent surgical removal of ingested magnets at our hospital over a period of 6 years. Additionally, we reviewed the literature to elucidate contemporary trends and optimal procedures for managing these patients, with an emphasis on surgical indications and outcomes in children.

## 2. Materials and methods

From January 2018 through December 2023, a total of 982 patients under the age of 17 were admitted to our hospital after ingesting foreign objects. Among these patients, 68 (6.9%) were specifically evaluated for the ingestion of magnets. Of these, 52 (76.5%) underwent removal via endoscopic intervention or were managed through passive monitoring, while 16 (23.5%) required surgical intervention.

We conducted a retrospective review of the medical records of the 16 pediatric patients who ingested magnets and subsequently underwent surgical removal at the Department of Pediatric Surgery, Kyungpook National University Hospital. We reviewed each patient’s demographic information, whether the magnet ingestion was witnessed, the interval between ingestion and admission, clinical presentation, the diagnostic techniques employed, the type and number of ingested magnets, the injury location, details of the surgical intervention, and postoperative outcomes.

The study protocol was approved by the Institutional Review Board of Kyungpook National University Hospital (IRB No. 2024-03-009). The requirement for informed consent was waived because this retrospective study used only existing, de-identified data without any personally identifying information.

## 3. Results

### 3.1. Demographic characteristics and clinical presentation

The study comprised a total of 16 pediatric patients (11 boys and 5 girls, yielding a male-to-female ratio of 2.2:1). The median age was 3.6 years, with an age range of 1 to 14 years. None of the patients exhibited developmental delay, intellectual impairment, or psychiatric disorders. The predominant presenting symptoms were abdominal pain (n = 6), abdominal pain accompanied by vomiting (n = 3), and a combination of fever, diarrhea, and seizure (n = 1). Six patients were asymptomatic.

The interval between magnet ingestion and hospital admission ranged from 6 hours to 2 weeks. One patient (Patient 7) with a history of surgical intervention for necrotizing enterocolitis during the second month of life was incidentally found to have ingested magnets on an abdominal X-ray during a routine outpatient follow-up.

Magnet ingestion was witnessed in only 4 of the 16 patients. Notably, every patient ingested at least 2 magnets, with the maximum number recorded being 24. The ingested magnets included 13 neodymium bead magnets, 2 round disc magnets, and one instance of a disc magnet accompanied by several iron parts. The comprehensive characteristics of these patients are summarized in Table 1.

**Table 1:** Demographic and clinical characteristics of the 16 patients who presented after magnet ingestion.

Patient no.	Sex	Age (years)	Clinical presentation	Witness to magnet ingestion	Duration from ingestion to admission	Preoperative diagnostic methods	Type of magnet
1	F	14	Asymptomatic	No	5 days	X-ray, CT, colonoscopy	Disc magnet
2	M	7	Abdominal pain	Yes	6 hours	X-ray, endoscopy	Neodymium bead magnet
3	F	5	Abdominal pain	No	2 weeks	X-ray, CT, colonoscopy	Disc magnet with iron piece
4	F	4	Asymptomatic	Yes	27 hours	X-ray, endoscopy	Neodymium bead magnet
5	M	1	Asymptomatic	Yes	16 hours	X-ray, CT, endoscopy	Neodymium bead magnet
6	M	2	Abdominal pain	No	10 hours	X-ray, CT	Neodymium bead magnet
7	M	1	Asymptomatic	No	N/A	X-ray	Neodymium bead magnet
8	M	3	Asymptomatic	Yes	10 days	X-ray, CT, endoscopy	Neodymium bead magnet
9	M	3	Abdominal pain	No	17 hours	X-ray, CT, endoscopy	Neodymium bead magnet
10	M	1	Abdominal pain	No	6 days	X-ray	Neodymium bead magnet
11	M	2	Abdominal pain, vomiting	No	2 days	X-ray, CT, endoscopy	Neodymium bead magnet
12	M	4	Asymptomatic	No	2 weeks	X-ray, CT, endoscopy	Neodymium bead magnet
13	F	8	Abdominal pain	No	28 hours	X-ray	Neodymium bead magnet
14	F	2	Abdominal pain, vomiting	No	8 hours	X-ray	Disc magnet
15	M	1	Abdominal pain, vomiting	No	7 hours	X-ray	Neodymium bead magnet
16	M	1	Fever, diarrhea, seizure	No	4 days	X-ray, CT, endoscopy	Neodymium bead magnet

CT = computed tomography, N/A = not available.

### 3.2. Preoperative diagnostic techniques

Preoperative diagnostic evaluation involved various imaging and endoscopic modalities. Eight patients underwent abdominal X-ray, endoscopy or colonoscopy, and abdominal computed tomography (CT); 5 patients were evaluated using abdominal X-ray alone; 2 patients underwent a combination of abdominal X-ray and endoscopy; and 1 patient was assessed using both abdominal X-ray and abdominal CT.

### 3.3. Surgical interventions and postoperative outcomes

All 16 patients required either laparoscopic exploration or laparotomy due to clinical deterioration or confirmed complications (such as perforation or obstruction) demonstrated on abdominal X-ray or CT scan. Fifteen patients initially underwent laparoscopic investigation; however, this approach proved technically challenging when the laparoscopic device became magnetically attached. Consequently, a minilaparotomy was performed for each of these patients via an additional incision adjacent to the umbilical trocar. One patient required primary laparotomy because of adhesions secondary to previous abdominal surgery. Table 2 summarizes surgical details and postoperative outcomes.

**Table 2:** Surgical details of the 16 patients who presented after magnet ingestion.

Patient no.	Location of magnet (number)	Operative methods	Hospital stays (days)	Morbidity and mortality
1	Ileum (3) to ileum (1)	Segmental resection of ileum, primary repair of ileum	8	No
2	Stomach (2) to jejunum (2)	Primary repair of stomach and jejunum	7	No
3	Transverse colon (21 metal) to jejunum (3)	Segmental resection of jejunum, primary repair of transverse colon	8	No
4	Gastroesophageal junction (2) to fundus (3)	Primary repair of stomach	8	No
5	Stomach (1) to jejunum (3) to jejunum (3)	Primary repair of jejunum	6	No
6	Jejunum (3) to jejunum (1)	Primary repair of jejunum	8	Intestinal obstruction
7	Stomach (7) to transverse colon (2)	Primary repair of stomach and transverse colon	14	Wound infection
8	Stomach (1) to transverse colon (1)	Primary repair of stomach and transverse colon	9	No
9	Stomach (1) to jejunum (15)	Segmental resection of jejunum, primary repair of stomach,	8	No
10	Ileum (2) to ileal mesentery (1)	Primary repair of ileum	7	No
11	Jejunum (1) to transverse colon (1)	Primary repair of jejunum and transverse colon	10	No
12	Stomach (3) to transverse colon (2)	Primary repair of stomach and transverse colon	8	Wound infection
13	Stomach (8) to transverse colon (2)	Primary repair of stomach	8	No
14	Stomach (7) to transverse colon (1)	Primary repair of stomach	7	No
15	Jejunum (3) to ileum (1) to ileal mesentery (1)	Primary repair of jejunum and ileum	9	No
16	Stomach (4) to transverse colon mesocolon (1) to jejunum (10)	Primary repair of stomach and jejunum	8	Death

The magnets were located in multiple sites, including between the stomach and the small intestine, as well as between the stomach and the transverse colon. For 13 patients, magnet extraction was performed with primary bowel repair, while for 3 patients, magnet extraction was accompanied by segmental resection followed by primary bowel repair. Postoperative hospital stays ranged from 6 to 14 days, with a mean duration of 8.1 days. Although 15 of the 16 patients experienced uneventful recoveries, 2 patients developed wound infections, and 1 patient (Patient 6) required adhesiolysis and segmental resection of the jejunum due to intestinal obstruction that occurred 10 months after the initial operation.

There was one death: a 1-year-old boy (Patient 16) who presented to the emergency room with a 4-day history of fever and convulsions. An abdominal X-ray revealed 15 magnets, and subsequent endoscopy identified only 4 magnets within the stomach. Given the patient’s unstable vital signs and suspicion of peritonitis, an exploratory laparotomy was performed. Eight days postoperatively, the patient died of septic shock. The 15 surviving patients were followed for a median of 24 months (range, 1–89 months) and are currently doing well.

## 4. Discussion

Children naturally tend to explore their environments by placing objects in their mouths, and their underdeveloped chewing and feeding skills often result in unintentional ingestion.^[1]^ Most unintentional FB ingestions occur between the ages of 6 months and 3 years (a period during which obtaining an accurate medical history may prove challenging).^[3,13]^ Studies have indicated that both sex and age significantly influence magnet ingestion, with boys being predominantly affected and approximately 50% of cases occurring in individuals under the age of 5 years.^[14,15]^ We observed a male-to-female ratio of 2.2:1, with 81.2% of patients being under 5 years of age, thereby corroborating previous reports.

Unwitnessed ingestion of magnets may only be identified after several days, at which point gastrointestinal injuries have already manifested. Magnet ingestion in children, particularly given the increased accessibility of high-powered magnets in toys and household items, has become as a significant pediatric emergency. Research in the United States has reported an 8.5-fold increase in emergency department visits related to magnet ingestion.^[16]^ Although the majority of FBs swallowed by young children transit the gastrointestinal tract spontaneously without causing harm, the risks associated with the ingestion of a single magnet differ markedly from those associated with ingesting multiple magnets. The ingestion of a single magnet is unlikely to cause gastrointestinal injury, and the magnet is typically expelled spontaneously. In contrast, ingesting multiple magnets individually or ingesting a single magnet in conjunction with other metallic foreign objects can result in their mutual attraction through the intestinal walls, leading to significant gastrointestinal damage (including pressure necrosis, fistula formation, bowel perforation, obstruction, and potentially peritonitis, and sepsis) that may necessitate urgent surgical intervention.^[11–13]^ Deaths resulting from the ingestion of multiple magnets have been documented; our study included a fatal case (Patient 16) that underscores the potential severity of these events.

Clinically, fewer than 40% of patients exhibit symptoms following magnet ingestion, with abdominal pain being the most common presentation. The patients in our study exhibited a variety of nonspecific clinical symptoms, including abdominal pain, vomiting, and fever. This heterogeneity in presentation underscores the diagnostic challenges associated with managing patients who have ingested magnets, as these symptoms may mimic other common pediatric conditions such as gastroenteritis or appendicitis. Abdominal symptoms typically manifest between 1 and 7 days following the ingestion of multiple magnets^[14,15]^; in our study, 75% of patients visited the emergency room within 1 week of ingestion. A substantial proportion of patients exhibited no peritoneal irritation despite the presence of intestinal perforation (a phenomenon likely attributable to the compartmentalization of intestinal leakage among adherent loops, thereby preventing widespread peritoneal contamination).^[15]^ These patients are particularly challenging to diagnose, as their symptoms (including vomiting, abdominal pain, diarrhea, and fever) closely resemble those of common influenza and other gastrointestinal disorders. This diagnostic ambiguity underscores the necessity of maintaining a high index of suspicion for magnet ingestion, particularly when young children present with unexplained abdominal pain. There are also instances wherein magnet ingestion is identified incidentally after an extended asymptomatic period. For example, we encountered a patient (Patient 7) who had previously undergone surgical intervention for necrotizing enterocolitis during the second month of life and was later found to have ingested magnets during a routine outpatient abdominal X-ray; these magnets were subsequently surgically removed.

Radiological imaging (namely, plain abdominal X-rays) serves as the primary modality for detecting swallowed metallic FBs or magnets. Once magnet ingestion is confirmed via X-ray, it is imperative to obtain multiple radiographic views to determine whether one or multiple magnets have been ingested or if a magnet has been ingested in conjunction with other metallic objects. This is particularly important given that magnets may adhere to each other, overlap on a single image, and masquerade as a solitary magnet. Moreover, the position and movement of the magnets within the gastrointestinal tract must be meticulously monitored to detect situations in which they exert pressure on adjacent bowel loops, potentially necessitating surgical intervention. Although the optimal interval between imaging studies remains inadequately defined, a recent National Patient Safety Alert recommends obtaining follow-up radiographs every 6 to 12 hours following the ingestion of highly powerful magnets.^[1]^ Bucci et al have advocated for the use of intestinal ultrasonography in cases of multiple magnet ingestions to identify any interposition of the intestinal wall between magnets located in separate loops.^[17]^ Supplementary imaging (such as CT scanning) is frequently necessary to assess for complications, including intestinal perforation.

Prompt identification and intervention are fundamental to the effective management of magnet ingestion incidents, even among asymptomatic individuals.^[18]^ Delayed diagnosis or treatment may lead to grave consequences, including intestinal necrosis, peritonitis, sepsis, and even death. Recent investigations into magnet ingestion have resulted in the development of a management algorithm for this scenario.^[19–22]^ When multiple magnets have been ingested, prompt endoscopic removal is indicated (provided the magnets have not passed beyond the pylorus). Once the magnets traverse the pylorus, some authors advocate for immediate surgical intervention, even in asymptomatic patients, to assess bowel viability,^[23]^ whereas others support delaying surgery until signs of peritoneal complications emerge.^[24]^ Generally, surgical removal of magnets is warranted when there is evidence of gastrointestinal perforation, obstruction, or clinical deterioration. In 2012, the North American Society for Pediatric Gastroenterology Hepatology and Nutrition established standards for managing swallowed foreign materials. According to their algorithm, management should be guided by the following variables: (1) the number of ingested magnets (single or multiple), (2) the site of impaction within the gastrointestinal tract (within or outside the stomach), (3) the presence or absence of symptoms, and (4) the timing of ingestion, if known. North American Society for Pediatric Gastroenterology Hepatology and Nutrition experts concur that if conservative management is chosen for patients with ingested magnets that have passed into the small bowel, direct surveillance in a controlled setting is essential. Furthermore, frequent abdominal radiographs and rigorous patient monitoring until confirmation of magnet passage are imperative. Luo et al developed a clinical nomogram that incorporates factors such as age, multiple ingestions, vomiting, abdominal pain, and abdominal tenderness to assist physicians in assessing the risk of complications arising from pediatric multiple magnet ingestion.^[25]^

Preventing magnet ingestion is a public health imperative. The increasing prevalence of small, high-powered magnets (often marketed as toys or novelty products) poses a significant hazard to children. It is essential that toys containing such magnets feature warning labels on their packaging to highlight the potential for serious injuries or life-threatening illnesses if ingested. Furthermore, public awareness campaigns, legislative measures to restrict the sale of these products, and enhanced education for both parents and healthcare practitioners are crucial to addressing the potential risks and complications associated with these seemingly innocuous objects.

## 5. Conclusions

During the history-taking process, it is essential to ask the patient’s parents and siblings whether any magnets are missing in cases of suspected magnet ingestion. If the ingestion of 2 or more magnets is suspected, immediate investigations should be initiated, including obtaining multiple-view X-rays, while avoiding the use of magnetic resonance imaging. If X-rays reveal the presence of magnets in the patient’s bowels, prompt intervention via endoscopic or surgical removal is advised to prevent complications. In conclusion, our study underscored the critical necessity for early diagnosis and swift surgical intervention in instances of magnet ingestion, especially when multiple magnets are involved. Timely identification, appropriate imaging, and prompt surgical intervention are essential to avert severe consequences. Enhanced education and preventive strategies (including public awareness initiatives regarding the hazards of small magnets) are crucial to reducing the frequency of these incidents. Collaboration among healthcare providers, regulatory agencies, and the public is vital to diminish the occurrence of magnet ingestion and improve outcomes for affected children. Pediatric surgeons should be involved promptly in complex cases, particularly those with multiple magnets that have traversed the stomach or in situations where there is a considerable delay between magnet ingestion and medical intervention.

## Author contributions

**Conceptualization:** Hyung Jun Kwon, Jinyoung Park.

**Data curation:** Hyung Jun Kwon, Jinyoung Park.

**Formal analysis:** Hyung Jun Kwon, Jinyoung Park.

**Writing – original draft:** Hyung Jun Kwon, Jinyoung Park.

**Writing – review & editing:** Hyung Jun Kwon, Jinyoung Park.
